# A mild aqueous synthesis of ligand-free copper nanoparticles for low temperature sintering nanopastes with nickel salt assistance

**DOI:** 10.1038/s41598-021-03707-9

**Published:** 2021-12-20

**Authors:** Hiroshi Imamura, Yoichi Kamikoriyama, Atsushi Muramatsu, Kiyoshi Kanie

**Affiliations:** 1 Institute of Multidisciplinary Research for Advanced Materials, Tohoku University, Sendai, 980-8577 Japan; 2 Business Creation Sector R&D Center, Mitsui Mining & Smelting Co., Ltd., Ageo, 362-0021 Japan

**Keywords:** Nanoparticles, Synthesis and processing, Electronic materials, Nanoparticle synthesis

## Abstract

An organic ligand-free aqueous-phase synthesis of copper (Cu) nanoparticles (NPs) under an air atmosphere was successfully achieved by reducing copper(II) oxide particles with a leaf-like shape in the presence of Ni salts at room temperature. The resulting Cu NPs with a mean particle diameter of *ca.* 150 nm exhibited low-temperature sintering properties due to their polycrystalline internal structure and ligand-free surface. These Cu NPs were applied to obtain Cu NP-based nanopastes with low-temperature sintering properties, and the resistivities of the obtained Cu electrodes after annealing at 150 °C and 200 °C for 30 min were 64 μΩ∙cm and 27 μΩ∙cm, respectively. The bonding strength between oxygen-free Cu plates prepared using the Cu NP-based nanopastes reached 32 MPa after pressure-less sintering at 260 °C for 30 min under a nitrogen atmosphere. The developed manufacturing processes using the developed Cu nanopastes could provide sustainable and low-CO_2_-emission approaches to obtain Cu electrodes on flexible films and high-strength bonding between metal plates as die-attach materials for power devices under energy- and resource-saving conditions.

## Introduction

Printed electronics (PE) technology has attracted a great deal of attention as one of the most promising manufacturing techniques for items such as electronic, mobile, and wearable future devices under low energy consumption and low carbon dioxide emission conditions; this technology is appealing because the electronic circuits and semiconductor layers in the devices are directly fabricated on the device substrates through a successive printing process under atmospheric conditions^1–3^. In PE technology, nano- and micron-sized metal particle inks and pastes are widely used to prepare conductive patterns on electronic circuit boards^4–8^. In particular, silver particle-based inks and pastes are the most representative and useful due to their advantageous characteristics, such as low resistivity, low-temperature sinterability, and high antioxidation ability^9^. Recently, these advantages have been applied in the combination of PE and integrated circuit (IC) production technologies, namely, flexible hybrid electronics (FHE) technology^10–12^. This technology enables us to fabricate flexible devices on plastic films with low thermal resistivity through an on-demand and eco-friendly process. To date, various types of future devices, such as wearable devices, have been developed and demonstrated based on market needs. Silver particle-based pastes have also recently received considerable attention as die-attach materials for the fabrication of next-generation SiC- and GaN-based power devices^13,14^. The larger bandgaps of SiC- and GaN-based power semiconductor materials than the corresponding Si-based materials enable device operation above 200 °C, which could permit miniaturization of the power modules. Furthermore, the high energy efficiency of high-temperature-driven power semiconductors is also quite suitable for the electrification of vehicles and is desirable for a future carbon–neutral society. As conventional die-attach materials, solder alloys have been widely used for Si-based device manufacturing. However, the low melting point and low thermal conductivity of solder alloys^15^ are becoming potential problems for die-attach materials from a viewpoint of application to high-temperature-driven SiC- and GaN-based power semiconductors^16^. To overcome these problems, low-temperature-sintered silver particle-based pastes with high thermal stability and high thermal conductivity have attracted much attention as promising candidates for die-attach materials applicable to SiC- and GaN-based device manufacturing. Although practical applications of silver particle-based inks and pastes are now rapidly progressing in PE, FHE, and power device technologies, fundamental problems of silver, such as its high cost as a precious metal and propensity for electromigration, inducing short circuits, are probably inescapable. More recently, copper (Cu) particle-based inks and pastes have rapidly been considered as candidates to solve silver-related problems in PE, FHE, and power device technologies because Cu is an inexpensive metal, has high electronic conductivity (1.68 µΩ·cm) and high thermal conductivity (398 W/m·K) values equivalent to those of silver and exhibits less electron migration than silver^17,18^. To date, liquid-phase synthesis of Cu nanoparticles (NPs) for ink and paste applications has been carried out in organic solvents in the presence of organic ligands to control the size and to prevent oxidation and aggregation of the Cu NPs^19,20^. However, synthesizing Cu NPs at a low cost on an industrial scale for practical applications is potentially problematic because the usage of organic solvents and organic ligands results in waste disposal issues. Furthermore, organic ligands, such as polymers and surfactants, remain on the surfaces of the particles, increasing the resistivity of the particles, and high-temperature treatment above 300 °C is required to remove these ligands from the surfaces of the particles to improve the resistivity and progress of sintering^21^. Such high-temperature sintering processes are difficult to apply in PE and FHE technologies because the flexible plastics used as substrates exhibit low thermal resistance^2^. Additionally, the development of a low-temperature die-attach process is desirable not only to reduce thermal damage to power semiconductor chips but also to save energy and resources. To date, photonic^22,23^, plasmatic^24^, and reducing atmospheric sintering^25,26^ have also been considered. However, these processes require expensive apparatuses and are not suitable for mass production on an industrial scale. To overcome this fundamental problem, Cu NPs with low-temperature sintering properties are desired, and such Cu NPs have recently been prepared by aqueous or polyol solvent systems in the presence of low-molecular-weight organic compounds^27–30^. Deng et al.^27^ reported that single nanosized Cu NPs were obtained in water in the presence of a large amount of glycolic acid or lactic acid. The resulting Cu NP-coated films exhibited values of 25.5 ± 8.0 µΩ·cm (glycolic acid) and 21.0 ± 7.0 µΩ·cm (lactic acid) after sintering at 150 °C under a N_2_ atmosphere. The utilization of organic amines with low boiling points has also been studied for Cu NP synthesis. Hokita et al.^28^ observed that the resistivity of 1-amino-2-propanol-modified Cu NPs reached 30 µΩ·cm after sintering at 150 °C under a N_2_ atmosphere. Mou et al.^29^ also applied 1-amino-2-propanol-modified Cu NPs to prepare a die-attach material for Cu–Cu bonding, and the shear and bonding strength reached 23 MPa upon heating at 200 °C under an argon atmosphere with 5 MPa pressure. The above described techniques enabled the development of low-temperature fabrication processes; however, a low oxidation resistance and low storage stability are fundamental problems for practical applications of these Cu NP-based pastes. More recently, we reported that polycrystalline Cu NPs were successfully obtained by reducing an aqueous copper(II)-nitrilotriacetic acid complex in a mild water system. The corresponding Cu nanopastes exhibited remarkable low-temperature sintering behaviour and high antioxidation properties and was applied to both fabricating electric circuits on flexible films and binding model IC chips on Cu substrates as power modules^30^. The process temperature was lower than 200 °C; however, it is difficult to further decrease the sintering temperature because the value strongly depends on the decomposition temperature of the organic compounds used for Cu NP synthesis. Furthermore, gas generation by the degradation of organic compounds by thermal treatment leads to a reduction in the electronic properties and bonding reliability of Cu electrodes and bonding materials because gas formation causes the formation of voids and cracks in the materials. In addition, using organic chemicals such as carboxylic acids and amines increases the manufacturing costs for Cu NPs.

Towards future material innovation in PE, FHE, and die-attach material technologies for a carbon–neutral society, the development of synthetic procedures for low-temperature sintering of Cu NPs without using organic chemicals has become an important issue to be resolved. Organic compounds are widely utilized to control the size and shape of NPs as well as to prevent the coagulation of NPs^31–34^. In this regard, a new concept to realize the size control of Cu NPs without the use of organic chemicals is urgently required. We focused on the addition of base transition metal salts for size control of Cu NPs because inorganic salts sometimes affect the size and shape of the resulting NPs^35,36^. The utilization of noble metal NPs is well known to accelerate heterogeneous nucleation to induce NP formation and growth^37–39^. However, using noble metals increases manufacturing costs. In this study, we report the aqueous-phase ambient synthesis of Cu NPs with low-temperature sinterability by the reduction of copper(II) oxide (CuO) particles with a leaf-like morphology in the presence of base transition metal salts without any usage of organic chemicals. The resulting Cu NPs were used to prepare a Cu NP-based nanopastes applicable not only for the fabrication of Cu electric circuits on flexible films but also as a die-attach material for the fabrication of next-generation power devices under mild sintering conditions, such as pressure-less and N_2_ atmosphere conditions.

## Results and discussion

### Preparation of CuO particles with a leaf-like shape for Cu NP synthesis

In liquid-phase synthetic systems of inorganic NPs based on nucleation followed by particle growth, the particle size can be controlled by complexation of the precursors with organic ligands because the initial nucleation number determining the final mean particle size obeys the solubility product of the resulting complexes in the system^40^. However, another method should be considered under organic ligand-free systems. In our previous studies, monodispersed copper(I) oxide (Cu_2_O) particles were successfully obtained by using CuO solid particles with a leaf-like morphology as the precursor in the presence of gelatine as an anti-aggregation agent^41^. The leaf-like shape with a wide specific surface area accelerates the dissolution rate of the CuO particles into the solvent; supersaturation and rapid nucleation are reached in a short period, resulting in highly monodispersed Cu_2_O particles. Kobayashi et al. also reported the synthesis of Cu particles using CuO solid particles as a raw material in cetyltrimethylammonium bromide (CTAB) aqueous solution^42^. In this case, a decrease in the mean particle diameter of the resulting Cu NPs was observed due to decreasing the particle size of the CuO precursors. This behaviour is also probably due to acceleration of the dissolution rate of the CuO precursors into the solvent by the size-dependent increase in the specific surface area of the CuO precursors. These previous studies suggested that utilization of solid precursors is a promising technique for ligand-free liquid-phase syntheses of NPs because controlling the dissolution rate of the solid precursors by controlling their shape and size leads to precise size control of purpose-designed NPs. In the present study, we focused on CuO solid particles^42^ with a leaf-like shape and therefore a large specific surface area as a raw material for the ligand-free synthesis of Cu NPs. The purpose-designed CuO particles were prepared as follows: initially, an aqueous light blue-coloured suspension of copper(II) hydroxides, which was obtained by mixing 5.0 L of 0.40 M Cu(NO_3_)_2_ and 5.0 L of 0.80 M NaOH aqueous solutions at room temperature, was aged at 40 °C for 8 h under atmospheric conditions. The solid particles thus obtained were filtered, washed with water, and dried at 120 °C prior to use for the present Cu NP synthesis.

**Figure 1:**
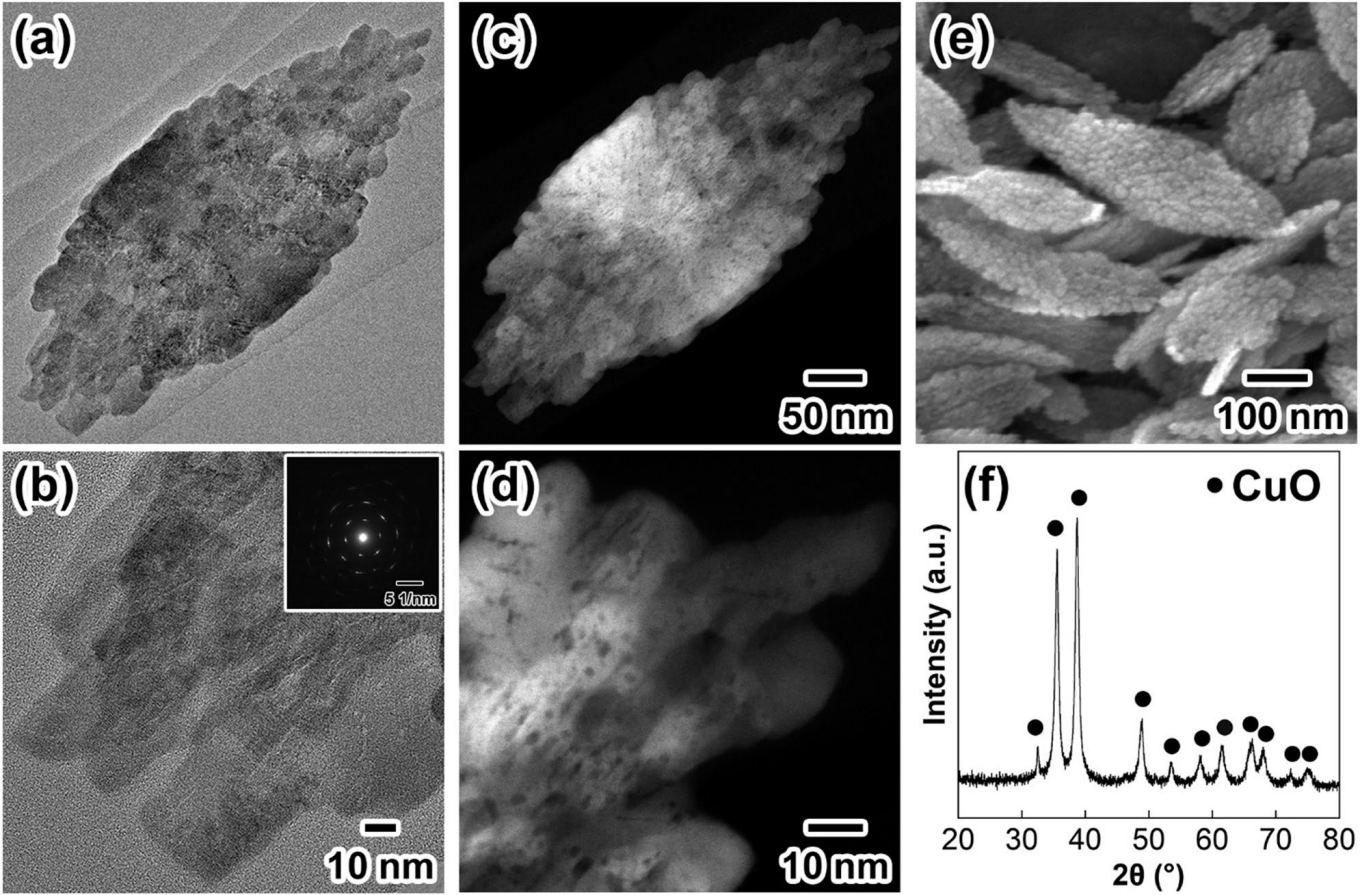
Results of characterization of CuO particles with a leaf-like shape as a solid precursor for Cu NP synthesis. (**a**) HR-TEM image; (**b**) magnified image of (**a**). The inset is the FT image of (**b**); (**c**) HAADF-STEM image; (**d**) magnified image of (**c**); (**e**) FE-SEM image; (**f**) XRD profile of the solid powder. The scale bar in (**b**) is the same as that for (**a**).

Figure 1 summarizes the characterization results for the solid particles used as the precursor for the preparation of Cu NPs. From the high-resolution transmission electron microscopy (HR-TEM) images shown in Fig. 1a,b, the particles consist of leaf-like shapes with a rough surface and internal voids. Similar polycrystalline images are also seen in the high-angle annular dark field scanning TEM (HAADF-STEM) images (Fig. 1c,d). The corresponding field emission scanning electron microscopy (FE-SEM) image in Fig. 1e reveals that the leaf-shaped particles have a major axis of 420 ± 89 nm, a minor axis of 245 ± 66 nm, and a thickness of several tens of nm. Small protrusions with a size of several tens of nm were observed on the surfaces of the particles in the SEM image. From the X-ray diffraction (XRD) pattern shown in Fig. 1f, all the diffraction peaks could be assigned to the formation of a single CuO crystal phase (JCPDS: 041-0254). The average crystallite size of the particles was calculated as 15 nm using Scherrer’s equation^43^ (Scherrer constant: 1.33), which was in good agreement with the primary particle size on the surface of the CuO particles in Fig. 1f. The results suggested that the CuO particles basically consist of a polycrystalline crystal structure. The inset in Fig. 1b presents a Fourier transform (FT) image of Fig. 1b. Highly oriented diffraction spots attributed to a single crystalline structure are visible in the FT images. These results mean that CuO particles with a polycrystalline structure were obtained through epitaxial growth of nuclei on the surface to produce primary particles with a crystallite size of *ca.* 15 nm. The specific surface area of the resulting polycrystalline particles was 22 m^2^/g, as determined by N_2_ adsorption measurements.

### Mild aqueous-phase synthesis of Cu NPs under organic ligand-free conditions by nickel salt assistance

As mentioned above, the addition of anti-aggregation agents such as gelatine^41^ and CTAB^42^ is a practical way to obtain highly monodispersed NPs. To establish an organic ligand-free liquid-phase synthetic method, we applied water‐soluble base transition metal salts during particle growth. The representative procedure for the preparation of Cu NPs in the presence of base transition metal salts is as follows: Initially, CuO particles (6.12 mmol, 0.487 g) with a leaf-like shape, NiCl_2_·6H_2_O (0.61 mmol, 0.148 g), and ion-exchanged water (20 mL) were mixed in a beaker (50 mL) with stirring (250 rpm) at room temperature under an air atmosphere. Then, an aqueous solution of hydrazine monohydrate (4.85 mol/L, 5.0 mL) was added in one portion to the mixture in the beaker with stirring (300 rpm) at room temperature. The resulting mixture was stirred for 2 h under the same conditions. The brown-coloured solids were collected by filtration using a cellulose acetate membrane filter with a pore size of 0.45 µm. The resulting solids were washed with ion-exchanged water until the conductivity of the filtrate became lower than 0.1 mS/cm. Finally, the collected solids were washed three times with denatured alcohol by centrifugation (10,000 G, 10 min) and dried under a reduced atmosphere to obtain Cu NPs. Further details of the synthetic procedure are summarized in the Methods section.

### Effects of nickel salt species and concentration on Cu NP synthesis

**Figure 2:**
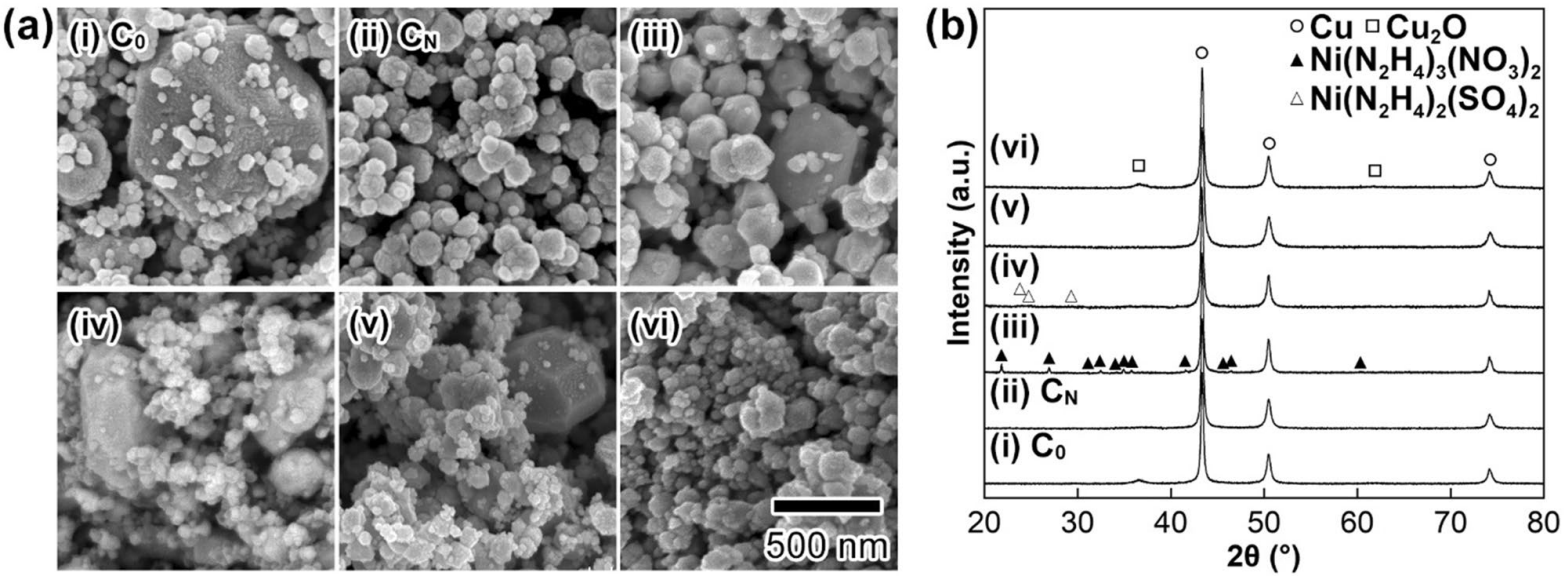
Results of morphological observation by FE-SEM and crystal structure analysis of Cu NPs by XRD. (**a**) FE-SEM images and (**b**) XRD patterns of the solid particles. (i): [Ni salt]/[CuO] = 0, **C**_**0**_; (ii): [NiCl_2_]/[CuO] = 0.10, **C**_**N**_; (iii): [Ni(NO_3_)_2_]/[CuO] = 0.10; (iv): [NiSO_4_]/[CuO] = 0.10; (v): [NiCl_2_]/[CuO] = 0.050; (vi): [NiCl_2_]/[CuO] = 0.20. The scale bar shown in (**a**)-(vi) is common to all images in (**a**).

Initially, we investigated the effects of nickel salt species on the size and shape of the resulting Cu NPs. Figure 2a-i exhibits an FE-SEM image of the solid particles obtained without the use of nickel salts (abbreviated as **C**_**0**_). Figure 2a-ii–iv show FE-SEM images of as-prepared particles in the presence of nickel chloride (NiCl_2_), nickel nitrate (Ni(NO_3_)_2_), and nickel sulfate (NiSO_4_), respectively. Here, the mixing molar ratio of Ni salts to CuO was fixed to 0.10 ([Ni salt]/[CuO] = 0.10). Both highly crystalline large particles with flat crystal planes (particle size: *ca.* 1 µm) and small NPs with an irregular shape (mean particle size: 144 ± 59 nm) were obtained when Cu NP synthesis was carried out without the use of Ni salts (Fig. 2a-i). In contrast, no highly crystalline particles were seen in the solid particles prepared in the presence of NiCl_2_ (Fig. 2a-ii), which are abbreviated as **C**_**N**_. This result suggests that NiCl_2_ has a positive effect on preventing the formation of highly crystalline large particles during the synthesis of Cu NPs starting from CuO. The effects of Ni(NO_3_)_2_ and NiSO_4_ on Cu NP synthesis were lower than that of NiCl_2_ because some highly crystalline large particles were clearly seen in Fig. 2a-iii,iv. The average particle sizes of the NPs, except for the large particles with flat crystal planes, obtained with NiCl_2_, Ni(NO_3_)_2_, and NiSO_4_ were 156 ± 48 nm, 209 ± 45 nm and 173 ± 77 nm, respectively. The particle size was determined by counting more than 200 NPs in the corresponding SEM images. Figure 2b shows XRD patterns of as-prepared particles in the absence or presence of nickel salts. The main diffraction peaks at 43.34°, 50.48°, and 74.14° could be assigned to the formation of a Cu metal phase as the main phase in the solid particles. The crystallite sizes of the Cu NPs obtained in the presence of NiCl_2_, Ni(NO_3_)_2_, and NiSO_4_ were calculated as 28 nm, 31 nm, and 39 nm, respectively^43^. The crystallite size was much smaller than the average particle size, indicating that the Cu NPs have a polycrystalline structure. Broad diffraction peaks due to a Cu_2_O phase in Fig. 2b suggest partial surface oxidation of the Cu NPs during purification. Similar behaviour was also observed in our previous study^30^. As shown in Fig. 2b-iii,iv, the formation of nickel hydrazine solid complexes was observed with the use of Ni(NO_3_)_2_ and NiSO_4_. Precipitation of such solid complexes is the most plausible reason why the effects of Ni(NO_3_)_2_ and NiSO_4_ on the prevention of highly crystalline large particles are lower than that of NiCl_2_ in the preparation of **C**_**N**_.

Next, we also tested the effect of the concentration of NiCl_2_ on Cu NP synthesis. Figure 2a-v,vi show FE-SEM images and XRD patterns of as-prepared Cu NPs with different [NiCl_2_]/[CuO] ratios of 0.050 and 0.20, respectively. The average Cu particle sizes of the samples were 246 nm and 123 nm. In the case of [NiCl_2_]/[CuO] = 0.050, highly crystalline large particles with flat surfaces (approximately 500 nm) were observed in the SEM images (Fig. 2a-v). No such particles were observed in the sample produced at [NiCl_2_]/[CuO] = 0.10: **C**_**N**_ and 0.20. The mean particle size of the Cu NPs decreased with increasing NiCl_2_ concentration. This result suggests that NiCl_2_ not only prevents the production of highly crystalline particles but also accelerates nucleation to obtain polycrystalline Cu NPs with small sizes. Such effects of NiCl_2_ on Cu NP synthesis might be largely due to a change in the reducing rate of N_2_H_4_ by complexation to form water-soluble NiCl_2_-N_2_H_4_ complexes to accelerate the nucleation number of Cu in the growth solutions. A change in colour from light green to light purple just after the addition of N_2_H_4_ to the NiCl_2_ aqueous solutions, suggesting complexation, was observed in the case of precipitation. Further mechanistic characterizations aiming to accelerate nucleation to obtain Cu NPs with the addition of NiCl_2_ are now in progress and will be reported elsewhere by the authors.

### Effect of metal salt species on Cu NP synthesis

**Figure 3:**
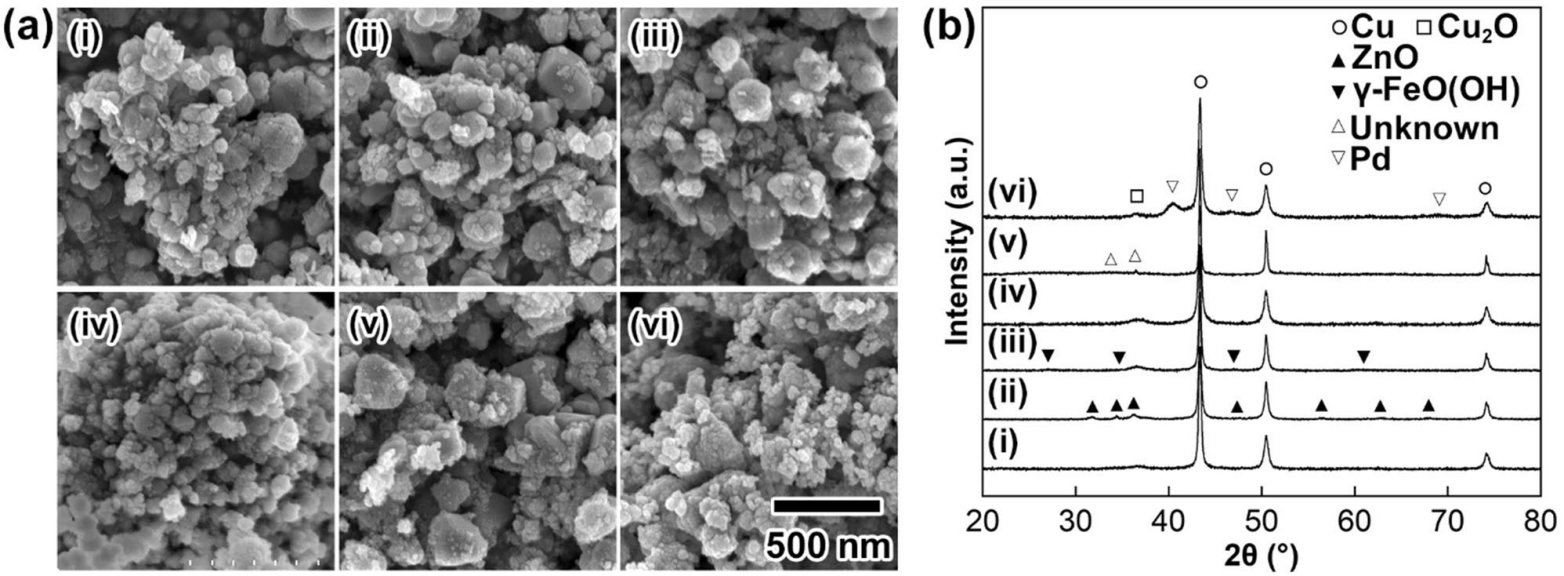
Morphological observation and crystal structure analysis of Cu NPs. (**a**) FE-SEM images and (**b**) XRD patterns of solid particles formed by changing the metal salt species: (i) CoCl_2_; (ii) ZnCl_2_; (iii) FeCl_2_; (iv) TiCl_4_; (v) SnCl_4_; (vi) PdCl_2_. The ratio of [metal salts]/[CuO] was fixed to 0.10. The scale bar shown in (**a**)-(vi) is the same for all images in (**a**).

Figure 3 exhibits the (a) FE-SEM images and (b) XRD profiles of solid particles obtained by adding (i) cobalt(II) chloride (CoCl_2_), (ii) zinc chloride (ZnCl_2_), (iii) iron(II) chloride (FeCl_2_), (iv) titanium chloride (TiCl_4_), (v) tin(IV) chloride (SnCl_4_), and (vi) palladium(II) chloride (PdCl_2_), instead of NiCl_2_, in the current Cu NP synthesis. All the synthetic conditions were the same as those for **C**_**N**_ except for the metal species. The [metal salt]/[CuO] ratio was fixed to 0.10 for all cases. The standard electrode reduction potentials of these metals are in the following order: palladium: 0.95 V; copper: 0.34 V; tin: − 0.14 V; nickel: − 0.26 V; cobalt: − 0.28 V; iron: − 0.76 V; zinc: − 0.76 V; titanium: − 1.63 V^44^. As shown in Fig. 3a-i–vi, the evolution of highly crystalline large particles with flat crystal planes, similar to those seen in Fig. 1a-i, was inhibited by the usage of metal chlorides. The addition of metal salts provably prevents uniform crystal growth, allowing the formation of highly crystalline Cu particles in the growth solutions. XRD measurements revealed that all the solid particles had a Cu metal phase as the main phase (Fig. 3b). The crystallite sizes of the Cu NPs in Fig. 3b-i–v were calculated as 30.8 nm, 38.8 nm, 43.9 nm, 25.3 nm, and 64.7 nm, respectively^43^. The crystallite sizes were smaller than the mean particle sizes and indicated polycrystalline structures for all particles. From the XRD profiles in Fig. 3b-ii,iii,v, ZnO, γ-FeOOH, and an unknown phase, respectively, contaminated the solid phases. Furthermore, amorphous-like precipitates attributable to titanium hydroxides are clearly seen in Fig. 3a-iv. Formation of these solid by-products in Cu NPs results in an increase in the resistivity of the material. When PdCl_2_ was used for the synthesis, a palladium metal phase was clearly observed (Fig. 3b-vi). The NPs a single nanometre in size in Fig. 3a-vi are considered palladium metal NPs. The higher redox potential of palladium than copper resulted in the formation of both palladium and copper metal NPs. These results suggested that the addition of metal chlorides such as NiCl_2_ and CoCl_2_ has a positive effect on obtaining Cu NPs for the synthesis of ligand-free Cu NPs applicable for Cu NP-based nanopastes with low-temperature sintering ability.

### Effects of reducing agents on Cu NP synthesis

**Figure 4:**
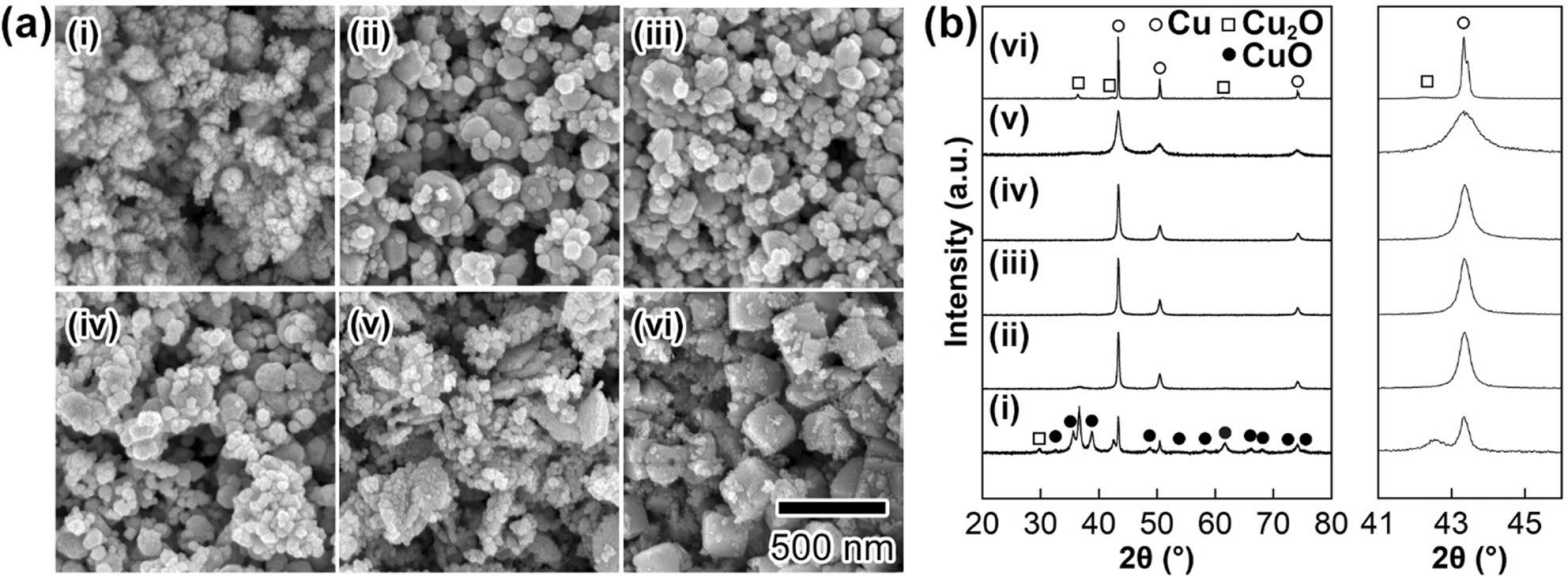
Results of (**a**) FE-SEM observations and (**b**) XRD measurements of solid particles formed by various ratios of [reducing agents]/[CuO]. Hydrazine, sodium borohydride (NaBH_4_), and ascorbic acid (AA) were chosen as the reducing agents. (i) [N_2_H_4_]/[CuO]: 1.0; (ii) [N_2_H_4_]/[CuO]: 2.0; (iii) [N_2_H_4_]/[CuO]: 8.0; (iv) [N_2_H_4_]/[CuO]: 16; (v) [NaBH_4_]/[CuO]: 4.0; (vi) [AA]/[CuO]: 4.0;. The scale bar shown in (**a**)-(vi) is common for all images in (**a**).

Figure 4a,b-i–iv show FE-SEM images and XRD patterns of solid particles obtained with different N_2_H_4_ concentrations ([N_2_H_4_]/[CuO]: (i) 1.0; (ii) 2.0; **C**_**N**_ 4.0; (iii) 8.0; (iv) 16). When [N_2_H_4_]/[CuO] was adjusted to 1.0, the reducing reaction for the formation of Cu NPs did not proceed perfectly, and the CuO phase used as the precursor was observed as the main phase (Fig. 3i). Upon increasing the [N_2_H_4_]/[CuO] ratio from 2.0 to 16, Cu NPs were formed in a single phase, and the mean particle size decreased with increasing N_2_H_4_ concentration. Figure 4v,vi show (a) FE-SEM images and (b) XRD patterns of solid particles obtained by using sodium borohydride (NaBH_4_) and l-(+)-ascorbic acid (AA) as reducing agents with [reducing agent]/[CuO] = 4.0. Some leaf-like particles assigned as Cu NPs are seen in Fig. 4a-v, suggesting that direct reduction from CuO particles with a leaf-like shape to corresponding Cu particles proceeded by using NaBH_4_. From the XRD profile in Fig. 4b-v, the crystallite size of the Cu particles was 15 nm, which further supported the direct reduction of the CuO particles because the crystallite size of the CuO particles was also 15 nm. This behaviour is probably due to the higher reduction ability of NaBH_4_ than N_2_H_4_. When AA was used as the reducing agent, Cu NPs with a cubic shape were obtained as the main product (Fig. 4vi). The crystallite size of 91.3 nm, calculated by using the XRD profile shown in Fig. 4b-vi, is similar to the corresponding mean particle size of the Cu nanocubes. It can be considered that the lower reduction ability of AA than N_2_H_4_ leads to the formation of single-crystalline Cu nanocubes by slow and uniform particle growth in the solvent. As described above for the optimization of the reaction conditions, we could conclude that NiCl_2_ and N_2_H_4_ are the most suitable compounds for Ni salt-assisted Cu NP synthesis for the development of Cu NP-based nanopastes with low-temperature sintering properties.

### Characterization of the surface and internal structures of C_N_

**Figure 5:**
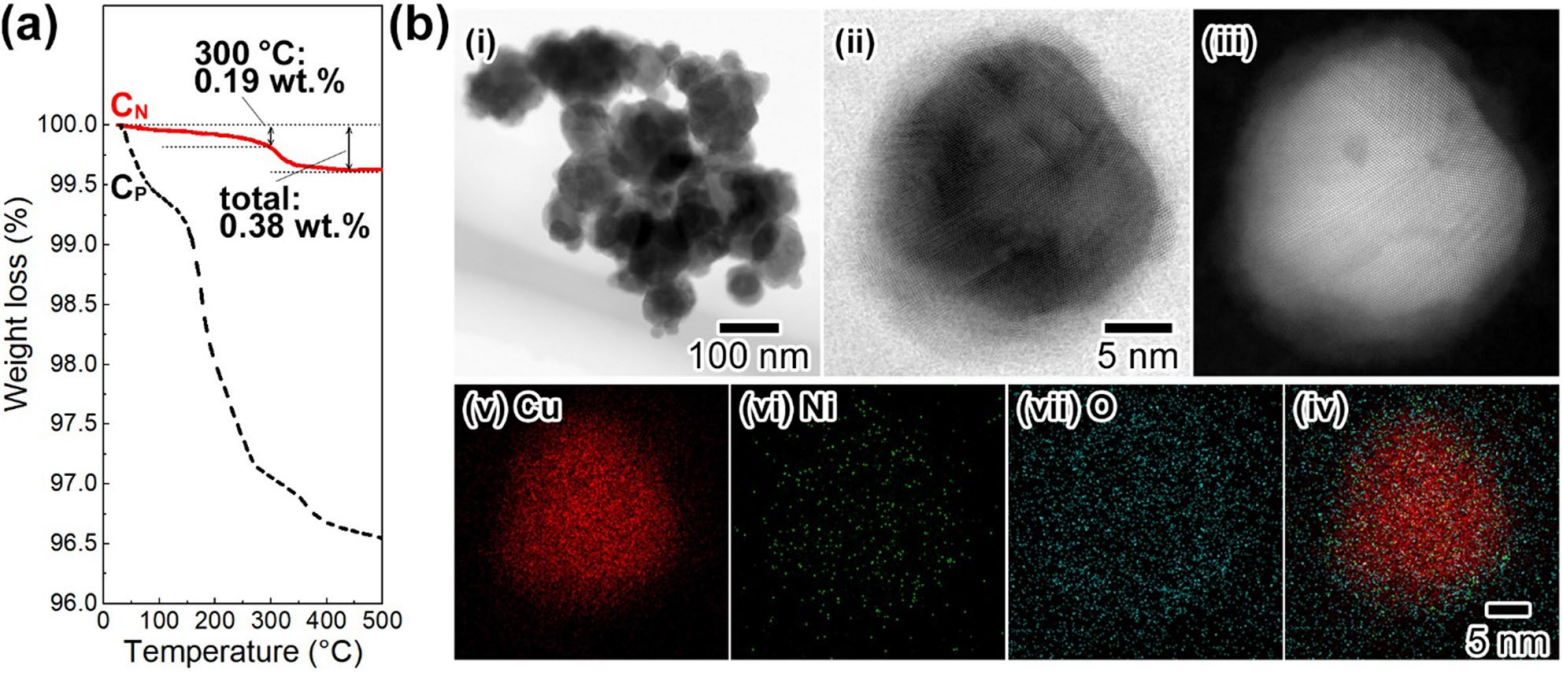
Results of characterization of **C**_**N**_. (**a**) TG profiles of **C**_**N**_ (a solid line) and **C**_**P**_^30^ (a dashed line). (**b**) (i) TEM image; (ii) HR-TEM image; (iii) HAADF-STEM image of the EDS analysis area. EDS images of (iv) copper *K*_α1_; (v) nickel *K*_α1_; (vi) oxygen *K*_α1_. (vii) Merged image of (iv–vi).

Figure 5a shows thermogravimetric (TG) profiles of **C**_**N**_ and our previously prepared Cu NPs using nitrilotriacetic acid (**C**_**P**_)^30^. Interestingly, the weight loss of **C**_**N**_ up to 300 °C was only 0.19 wt%. Upon further heating, a 0.38 wt% weight loss was observed at approximately 310 °C for **C**_**N,**_ which is due to decomposition of a Cu_2_O phase on the surface of **C**_**N**_. In contrast, the weight loss of **C**_**P**_ up to 300 °C was 2.9 wt%. The loss is due to degradation of organic residues on the surface of **C**_**P**_. Such degradation has a potential problem to lead to gas generation in the preparation of Cu electrodes and adhesion of IC chips with the usage of Cu NP-based nanopastes. From this point of view, the present **C**_**N**_ obtained under Ni salt-assisted ligand-free conditions has a large potential for application in Cu NP-based nanopastes with minimal gas evolution. Figure 5b shows the observed internal structures and surface states of **C**_**N**_ obtained by TEM and HAADF-STEM equipped with an energy dispersive X-ray spectroscopy (EDS) system. The polycrystalline crystal structure of **C**_**N**_ is clearly seen in the TEM image in Fig. 5b-i. The HR-TEM image suggests the existence of a rough shell structure on the surface of **C**_**N**_ with a thickness of *ca.* 3 nm (Fig. 5b-ii). From the XRD profile of **C**_**N**_ in Fig. 2b-ii, **C**_**N**_ contains a Cu_2_O phase. This result means that the shell consists of a Cu_2_O phase due to the partial surface oxidation of **C**_**N**_ during the purification process. Figure 5b-iii is the HAADF-STEM image of **C**_**N**_. The corresponding EDS mapping patterns of copper, nickel, and oxygen and the merged image are seen in Fig. 5b-iv–vii, respectively. These images suggest that Ni atoms are incorporated and uniformly distributed in **C**_**N**_ and oxygen atoms are distributed on the surface of **C**_**N**_ to form the Cu_2_O shell layer. X-ray photoelectron spectroscopy (XPS) revealed that Ni species on the surface of Cu particles exist mainly as oxides. The polycrystalline core–shell structure of **C**_**N**_ provides a low sintering temperature, high oxidation resistance, and long-term stability.

### Sintering behaviour of Cu NP-based pastes

**Figure 6:**
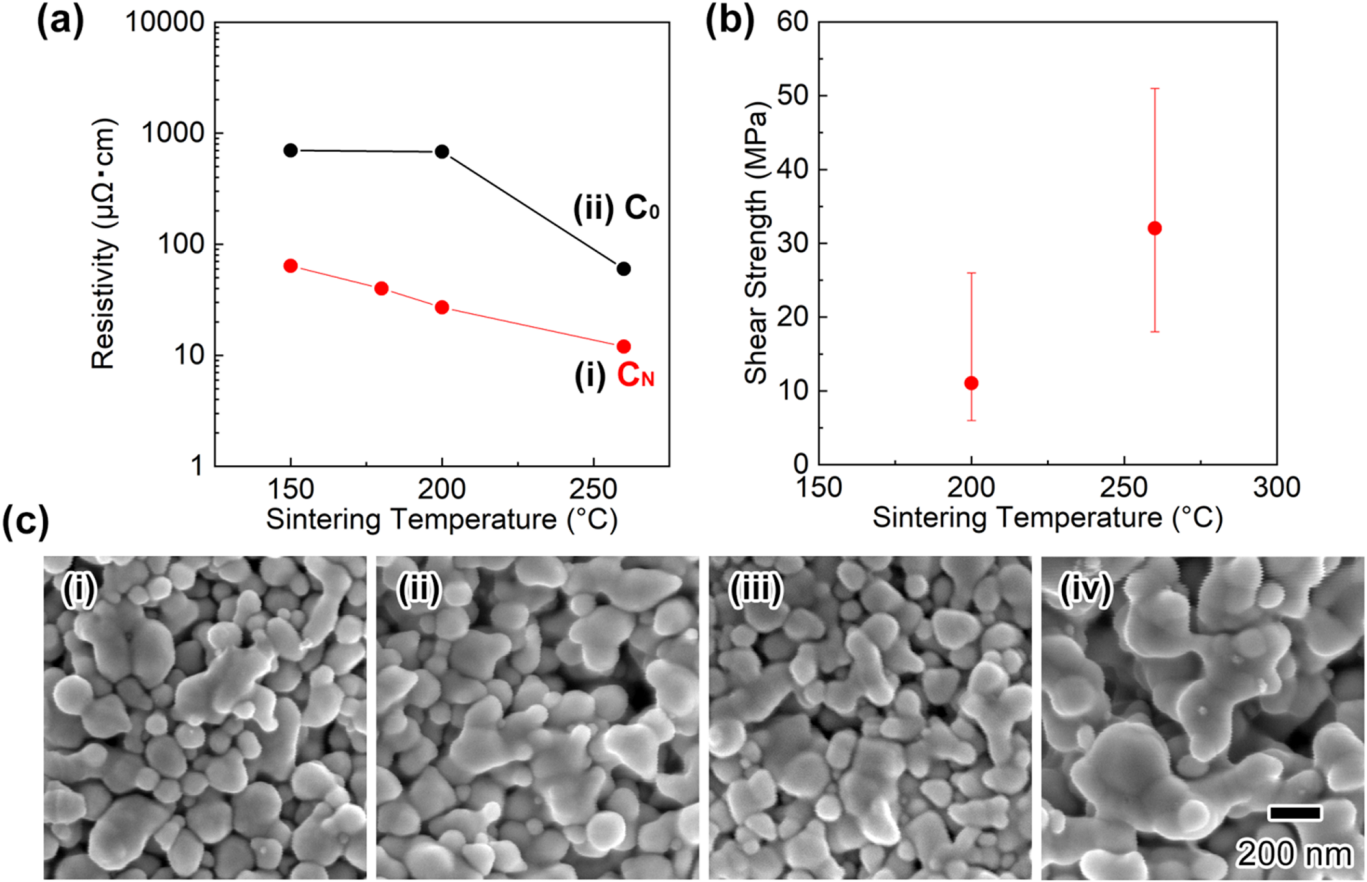
Sintering behaviour of the NP paste and characterization of sintering films. (**a**) Resistivity of sintering films at different temperatures made from pastes of copper particles synthesized with different molar ratios of [NiCl_2_]/[Cu] (i): 0.1 (Ni-salt-assisted method), (ii): 0. (**b**) Shear strength of NP pastes obtained at different temperatures by pressure-less sintering under a N_2_ atmosphere. (**c**) SEM images of Cu sintering films made from nanopastes on a glass substrate sintered at (i) 150 °C, (ii) 180 °C, (iii) 200 °C, and (iv) 260 °C under a N_2_ atmosphere for 30 min.

Figure 6a summarizes the resistivity of Cu electrodes fabricated by using Cu NP-based nanopastes with different sintering temperatures. (i) **C**_**N**_ and (ii) **C**_**0**_ were chosen as the Cu NPs to evaluate the effect of Ni salt assistance on the sintering and electric properties. The resulting resistivities of Cu electrodes fabricated with **C**_**N**_-based nanopastes at sintering temperatures of 150 °C, 180 °C, 200 °C, and 260 °C were 64 µΩ·cm, 40 µΩ·cm, 27 µΩ·cm, and 12 µΩ·cm, respectively, which were lower than those of **C**_**0**_-based electrodes (cf. the resistivities of electrodes sintered at 150 °C, 200 °C, and 260 °C were 700 µΩ·cm, 681 µΩ·cm, and 60 µΩ·cm, respectively). Figure 6b exhibits the shear strength of Cu–Cu bonded bodies, representing a model IC chip, adhered by using the **C**_**N**_-based nanopastes under pressure-less and N_2_ atmospheric conditions. The details for the preparation of the model IC chips are summarized in the Methods section below. The shear strengths after sintering at 200 °C and 260 °C for 30 min were 11 MPa and 32 MPa, respectively. A shear strength greater than 30 MPa at 260 °C under pressure-less conditions is suitable for practical applications to produce power devices^30^. Figure 6c shows FE-SEM images of sintered Cu electrodes prepared by using the **C**_**N**_-based nanopastes. From the images, sintering between **C**_**N**_ NPs and the formation of necking structures was already underway at 150 °C, and the growth of necking structures proceeded upon increasing the sintering temperature from 150 to 260 °C (Fig. 6c-i–iv). The crystallite sizes of the Cu electrodes after sintering at 150 °C, 180 °C, 200 °C, and 260 °C were 41.5, 47.1, 55.5, and 69.4 nm, as determined by XRD measurements. The tough Cu–Cu bonding is due to the sintering process between Cu NPs and associated formation of necking structures under mild conditions.

### Preparation of copper electrodes on flexible films from Cu NP-based nanopastes

**Figure 7:**
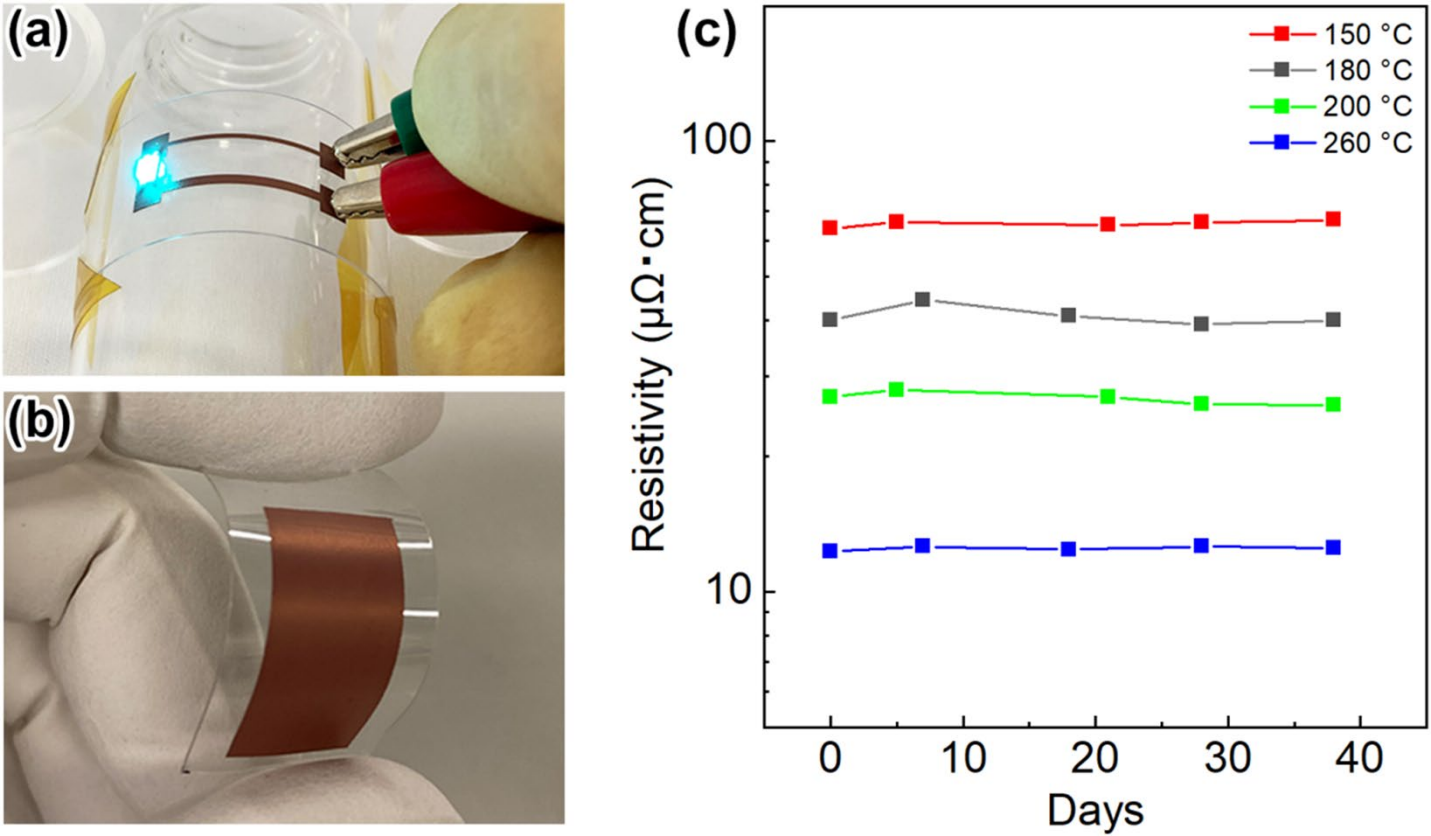
Characteristics of copper electrode formation on flexible substrates and stability of the copper electrode. (**a**) LED-mounted Cu electrode obtained by printing **C**_**N**_ NP-based nanopastes on a PEN film and the LED emission behaviour. The device was prepared by sintering at 200 °C under a N_2_ atmosphere for 30 min. (**b**) A **C**_**N**_ NP-based electrode on a PET film obtained after sintering at 150 °C under a N_2_ atmosphere for 30 min. (**c**) Time changes in the resistivity of Cu electrodes obtained by sintering at 150 °C, 180 °C, 200 °C, and 260 °C under a N_2_ atmosphere for 30 min. The Cu electrodes on a glass substrate were kept under ambient atmospheric conditions at 24 ± 1 °C and 44 ± 1% humidity, and the resulting resistivities were measured for the stability test.

Figure 7a,b show photographs of Cu electrodes on a polyethylene naphthalate (PEN) film and a polyethylene terephthalate (PET) film, respectively, prepared by using **C**_**N**_ NP-based nanopastes. The sintering temperatures for Fig. 7a,b were 150 °C and 200 °C, respectively. Details for the preparation are summarized in the Methods section. No cracks were seen after repeated bending of the Cu electrode printed flexible films, and a simple light emission diode (LED) device on a PEN film was readily obtained. Figure 7c shows the results of stability testing of the Cu electrodes prepared by sintering at 150 °C, 180 °C, 200 °C, and 260 °C under a N_2_ atmosphere for 30 min. After preparation of the Cu electrodes on a PEN film, the electrodes were left under atmospheric conditions at a temperature of 24 ± 1 °C and humidity of 44 ± 1% for the stability test. Regardless of the sintering temperature, the resistance of the Cu electrodes remained almost unchanged for more than one month.

## Conclusions

In the present study, we have established an organic ligand-free aqueous-phase synthesis of Cu NPs that are applicable for use in Cu NP-based nanopastes with low-temperature sintering properties. Here, the addition of NiCl_2_ played a critical role in reducing CuO solid particles with a leaf-like shape by hydrazine to obtain **C**_**N**_ from an aqueous phase under ambient atmospheric conditions. Interestingly, the weight loss of **C**_**N**_ due to organic residues on its surface was only 0.19 wt%, as determined from TG–DTA measurements. The results suggested that **C**_**N**_ with an organic ligand-free surface obtained by Ni salt assistance is a promising material for the development of Cu NP-based nanopastes with minimal gas evolution during Cu electrode fabrication and Cu–Cu adhesion. Such gas formation is a serious problem in PE and FHE technologies and leads to the generation of cracks and voids, which reduce the quality and durability of the products. Due to the unique polycrystalline core–shell structure of **C**_**N**_, determined by XRD, TEM, and HAADF-STEM characterizations, **C**_**N**_ NP-based nanopastes with low-temperature sintering and antioxidation properties under atmospheric conditions were successfully obtained and applied for the fabrication of Cu electrodes on flexible films. The resistivity of Cu electrodes on a flexible film reached 64 µΩ·cm and 27 µΩ·cm after sintering at 150 °C and 200 °C, respectively. Furthermore, as a model of IC chip adhesion, the formation of Cu–Cu bonded bodies with a high shear strength of 32 MPa was achieved with the use of the Cu NP-based nanopastes after pressure-less sintering under a nitrogen atmosphere. The present Cu NP-based nanopastes are a promising material for achieving sustainable progress in PE, FHE and power device technologies and are expected to be applied in next-generation on-demand fabrication processes^45^ for future smart products, such as wearable devices, under energy- and resource-saving conditions.

## Method

### Reagents

Unless otherwise noted, all reagents were used as received without further purification. Water was doubly distilled, deionized, and filtered prior to use. Copper nitrate trihydrate (Cu(NO_3_)_2_∙3H_2_O, Cat. No: 037-12505), sodium hydroxide (NaOH, Cat. No: 194-18865), nickel chloride hexahydrate (NiCl_2_∙6H_2_O, Cat. No: 141-01045), nickel nitrate hexahydrate (Ni(NO_3_)_2_∙6H_2_O, Cat. No: 147-01101), nickel sulfate hexahydrate (NiSO_4_∙6H_2_O, Cat. No: 146-01171), cobalt chloride hexahydrate (CoCl_2_∙6H_2_O, Cat. No: 036-03682), zinc chloride (ZnCl_2_, Cat. No: 263-00271), iron chloride tetrahydrate (FeCl_2_∙4H_2_O, Cat. No: 099-00915), titanium chloride (TiCl_4_, Cat. No: 202-12592), palladium(II) sodium chloride trihydrate (PdCl_2_∙2NaCl∙3H_2_O, Cat. No: 167-00081), sodium tetrahydroborate (NaBH_4_, Cat. No: 195-11455), and l-(+)-ascorbic acid (C_6_H_8_O_6_, Cat. No: 016–04,805) were purchased from FUJIFILM Wako Pure Chemical Corporation. Tin chloride (SnCl_4_, anhydrous, Cat. No: 37312-01), hydrazine monohydrate (N_2_H_4_∙H_2_O, Cat. No: 18383-00), triethanolamine (N(CH_2_CH_2_OH)_3_, Cat. No: 40268-08), and dehydrated methanol (CH_3_OH, Cat. No: 25506-25) were purchased from Kanto Chemical Co., Inc. 3-Glycidoxypropyltrimethoxysilane (Cat. No: KBM-403) was purchased from Shin-Etsu Chemical Co., Ltd. Denatured alcohol was purchased from IMAZU CHEMICAL Co. Ltd.

### Preparation of CuO particles as a precursor for Cu NP synthesis

Initially, 0.40 M Cu(NO_3_)_2_ (5.0 L) and 0.80 M NaOH (5.0 L) aqueous solutions were mixed together at room temperature with stirring. Then, the resulting slurry was further stirred at 40 °C for 8 h. During this stage, the colour of the slurry changed from light blue to brown. The brown-coloured solids thus obtained were collected by filtration and washed with ion-exchanged water by centrifugation (20,000 G, 30 min) until the conductivity of the supernatant liquid became lower than 0.1 mS/cm. Finally, the solids were dried at 120 °C in a fine oven to obtain CuO particles with a leaf-like shape.

### Metal salt-assisted liquid-phase synthesis of Cu NPs

Self-prepared CuO particles with a leaf-like shape (6.12 mmol, 0.477 g), metal salts (0.61 mmol), and ion-exchanged water (20 mL) were added to a beaker and mixed with stirring (250 rpm) at room temperature under an air atmosphere. Then, an aqueous solution of 4.85 M hydrazine monohydrate (24.3 mmol, 5.0 mL) was added to the mixture in the beaker in one portion with stirring (250 rpm) at room temperature. The mixture was stirred for 2 h under the same conditions. The resulting solid particles were collected by filtration using a cellulose acetate membrane filter (pore size: 0.45 µm) and washed with deionized water until the conductivity of the filtrate was under 0.1 mS/cm. Then, the particles were washed three times with denatured alcohol by dispersal and centrifugation (10,000 G, 10 min), and the Cu NPs were collected as an ethanol slurry (70 wt% Cu). The slurry was dried under reduced pressure to obtain Cu NPs as a powder for characterization. The effect of NiCl_2_ concentration on Cu NP preparation was examined by changing the addition amount of NiCl_2_ to 0, 0.31, and 1.2 mmol ([metal salt]/[Cu]: 0, 0.050, and 0.20). To investigate the effect of hydrazine concentration on Cu NP synthesis, the aqueous solution concentration of 4.85 M hydrazine monohydrate was changed to 1.21 M, 2.43 M, 9.7 M, and 19.4 M ([N_2_H_4_]/[Cu]: 1.0, 2.0, 8.0, and 16).

### Preparation of Cu NP-based nanopastes

For the preparation of the **C**_**N**_ NP-based nanopastes, an ethanol slurry of **C**_**N**_ (70 wt%) was synthesized at a scale 20 times larger than that described above (the stirring time after the addition of the aqueous solution of hydrazine monohydrate was 1 h). Then, the resulting slurry was dried in a reduced‐pressure atmosphere, and the obtained solid particles were ground with an agate mortar in a N_2_ atmosphere and sieved to 635 mesh. Next, **C**_**N**_ (2.00 g) and triethanolamine (0.35 g) were mixed in a N_2_ atmosphere using a rotation-revolution mixer (ARE-310, THINKY CORPORATION) and sieved to 635 mesh. Finally, 0.081 g methanolic solution of 3-glycidoxypropyltrimethoxysilane (63 wt%) was added to the mixture (1.00 g) to obtain the **C**_**N**_-based nanopastes (79 wt% **C**_**N**_).

### Preparation of Cu NP-based patterned electrodes on glass substrates

The Cu NP-based pastes were printed on a glass substrate (30 mm × 15 mm, OA-10, Nippon Electric Glass Co., Ltd.) with a size of 20 mm × 10 mm by screen printing. The thickness of the pastes was adjusted to 55 μm. Then, the printed glass substrate was pressure-less sintered at 150 °C, 180 °C, 200 °C, and 260 °C for 30 min under N_2_ flow (1.3 L/min). The resistivity of the Cu electrodes obtained by sintering at the different temperatures was measured by a four-point probe method.

### Fabrication of Cu NP-based patterned electrodes on flexible films

A PEN film (Teonex Q65FA, TEIJIN Ltd., *t* = 200 μm) and a PET film (DUORA, SEKISUI CHEMICAL CO., LTD., *t* = 160 μm) were used as the flexible films. Model Cu electrodes were fabricated on the PEN film by screen printing the Cu NP-based paste using a screen mask (high-density mesh ST 500, emulsion thickness: 15 μm, Tokyo Process Service Co., Ltd.) and then sintered at 200 °C for 30 min under a N_2_ flow (1.3 L/min). The Cu electrode on a PET film was prepared using the Cu NP-based paste by screen printing with a size of 20 mm × 10 mm. Then, the printed film was pressure-less sintered at 150 °C for 30 min under a N_2_ flow (1.3 L/min) to obtain Cu electrodes on the film.

### Preparation of copper-bonded bodies and shear strength measurements

Flat Cu plates with a clean surface were prepared by rotary polishing oxygen-free Cu plates (i-ject Co., Ltd., 3 × 3 mm and 5 × 5 mm; *t* = 1 mm) with a manual polishing machine (grit size: # 4000). Then, the resulting Cu plate (5 × 5 mm) on which the Cu NP-based pastes (1 mm square) were printed was prepared by screen printing using a metal mask (Tokyo Process Service Co., Ltd.). Then, the 3 × 3 mm plate was placed on the printed nanopaste, and the paste was sandwiched between the Cu plates. The resulting Cu plates were sintered at 200 °C and 260 °C for 30 min under pressure-less conditions with flowing N_2_ (1.3 L/min) for adhesion. The shear strength of the resulting Cu-bonded bodies was measured and calculated by a bond tester (Condor Sigma, XYZTEC) at a shear speed of 50 μm/s.

### Characterization equipment

X‐ray diffraction (XRD) measurements were performed on a Rigaku Intelligent X-ray diffraction SmartLab system equipped with a PILATUS3 R 100 K detector using CuKα radiation (40 kV, 40 mA) to determine the crystal phase and measure crystallite size. Scanning electron microscopy (SEM) observations were carried out using a HITACHI SU 7000 with an acceleration voltage of 5 kV. TEM observations were performed using an FEI TITAN 80–300 instrument at 200 kV to obtain HR-TEM and high-angle annular dark field scanning TEM (HAADF-STEM) images. The electrical conductivity of the copper electrodes was measured by a Mitsubishi Chemical Analytech Loresta-GX MCP-T 700 instrument with a four-point probe method. Thermogravimetric analysis (TGA) of the Cu NPs was carried out by a Bruker Japan TG-DTA2000SA at a heating rate of 5 °C/min under atmospheric conditions.
